# Cyclic Vomiting Syndrome in a Male Patient With History of Renal Cell Carcinoma Status Post Nephrectomy

**DOI:** 10.4021/jocmr2009.12.1278

**Published:** 2010-02-26

**Authors:** Navid Ezra, Sonal Patel, Mehran Kashefi

**Affiliations:** a David Geffen School of Medicine at UCLA, Los Angeles, USA; b Department of Medicine, Cedars-Sinai Medical Center, Los Angeles, USA; c Department of Medicine, Veterans Administration Greater Los Angeles Healthcare System, Los Angeles, USA

## Abstract

Cyclic vomiting syndrome (CVS) is a condition whose symptoms are recurring attacks of intense nausea, vomiting and sometimes abdominal pain and/or headaches or migraines. Cyclic vomiting usually develops during the childhood ages of 3 - 7; whereas it often remits during adolescence, it can persist into adult life. We report a case of a 57 years old Caucasian male with a history of renal cell carcinoma status post left nephrectomy presenting with a 7 years history of intermittent nausea and vomiting with prodromal malaise, watery diarrhea, flushing and diaphoresis. Cyclic vomiting syndrome is a debilitating functional brain-gut disorder that was initially characterized in children, but now is increasingly recognized to occur also in adults.

**Keywords:**

Cyclic vomiting syndrome; Vomitting; Post nephrectomy

## Introduction

Cyclic vomiting syndrome (CVS) is an overlooked but common functional disorder commonly diagnosed in childhood, consisting in paroxysms of severe nausea and vomiting separated by symptom free periods [1-3]. CVS is increasingly recognized in adults with recent research providing a framework for management and further investigations into CVS in adults [4].

## Case Report

A 57 years old Caucasian male presented with recurrent nausea, vomiting, abdominal pain and diarrhea for the past 1 - 2 years. Patient stated he had multiple episodes of retching and bilious vomiting in the past 24 hours, with one episode of hematemesis 2 hours before coming to the emergency department. He had no prior episodes of hematemesis.

He described his abdominal pain as diffuse, was occasionally sharp initially, with residual dullness which slowly resolved over 24 - 36 hours. The pain was not associated with food, and not relieved or worsened with bowel movements. His stool had been of regular caliber, color and consistency, and he had approximately 1 bowel movement daily.

His history dated back approximately twenty years ago when he started complaining of dyspepsia. He was treated at an outside hospital for H. Pylori, and remained relatively well controlled until seven years prior to admission when he started to notice discrete episodes of malaise, followed by 1 day of loose watery stools, then another day of intense bouts of bilious but non bloody emesis (up to 30 episodes a day) which would leave him with (usually) dull abdominal pain that gradually resolved over the next 2 days.

Besides associated flushing and diaphoresis with the diarrhea, he could not state any triggers or associated symptoms. He endorsed a 20 lb weight loss in the past year. No constitutional symptoms, such as heartburn, dysphasia, odynophagia, bright red blood per rectum or steatorrhea were found.

His past medical history was significant for an incidental discovery of a renal mass on MRI (magnetic resonance imaging) two years previous, with eventual left nephrectomy for renal cell carcinoma (RCC) soon after discovery. He also had a history of ‘gastroenteritis’, hyperlipidemia, and chronic back pain.

The patient’s medications included Omeprazole 20mg QDay, Gabapentin for CBP, Loperimide Q8 hours prn loose stools, Psyllium husk BID for constipation, and PRN vicodin for chronic back pain. He had no known drug allergies. Family history was pertinent for a mother with Crohn's disease and a sister with migraines. The patient denied tobacco, alcohol, and drug use.

On physical examination, patient’s vitals were normal and stable. He appeared as a thin male in no apparent distress, but looking fatigued. His abdomen was flat with a visible well-healed nephrectomy scar at the left lower quadrant. Bowel sounds were present in all 4 quadrants. All other parts of the physical exam were within normal parameters. Complete blood count, basic metabolic panel, liver function tests, and coagulation markers returned in the normal ranges. Urine analysis was clear with a pH of 7 and nitrite and leukocyte esterase negative.

Imaging included a CT (computed tomography) of the abdomen/pelvis on six months prior that was read with questionable findings of ‘probable wall thickening of colon and possible distal ileum, raising possibility of inflammatory bowel disease or an infectious process.’ Also a left renal lesion was seen.

An esophageal gastroduodenoscopy (EGD) on four months prior revealed a 5cm hiatal hernia, acid reflux esophagitis, and cobblestone appearance of stomach of unclear clinical significance. Biopsy results showed mucosa without significant pathological change in most of the stomach with minimal reactive gastropathy in the antrum.

Colonoscopy four months prior showed colonic mucosa without significant pathologic changes and lymphoid mucosa. A tubular adenoma polyp was seen at 40 cm of the colon, a tubular adenoma polyp at the cecum, and a rectal hyperplastic polyp. Histopathological features in these biopsies were not commensurate with a diagnosis of inflammatory bowel disease.

A current EGD showed a hiatal hernia with a Mallory Weiss tear. A Schatzki ring was present.

## Discussion

CVS has been described infrequently in adults [5,6]. The reported patient displayed clinical features that met the diagnostic criteria of CVS. Diagnosis is based on criteria established in the First International Scientific Symposium on CVS in 1994, from which CVS is defined by recurrent, severe, discrete episodes of vomiting with varying intervals of normal health between episodes that last from hours to days and no apparent cause of vomiting is found, with negative laboratory, radiographic and endoscopic test results [2].

The pathophysiology of CVS has not yet been established, and treatment for CVS remains unsatisfactory [4]. Mitochondrial dysfunction [7] or calcium ion channel aberration has been suggested in the etiology of CVS [8].

Treatment focuses on prevention of attacks, termination of vomiting, sedation and prevention of complications. The management of CVS is limited, and only a small number of maintenance therapies have so far been reported to be effective. Although treatment of CVS in both children and adults remains to the most part unsatisfactory, tricyclic antidepressant medication has been recommended as the standard therapy, with greater efficacy in children [6,9,10]. Ondansetron may be an eligible drug for faster control of the syndrome in adults [11].

The efficacy of an anticonvulsant for CVS has recently supported the existence of a nosological link between CVS, migraine, and epilepsy [10, 12-1410, 12-14]. Treatment with carbamazepine has also resulted in complete remission of the vomiting episodes [15].

Cyclic vomiting is a heterogeneous syndrome whose etiology is still unclear. Further investigations are needed to further elucidate the pathogenetic substrate and identify better therapeutic modalities.
